# Outcome measurements in epidermal necrolysis: a systematic review

**DOI:** 10.1007/s00403-024-03062-5

**Published:** 2024-06-15

**Authors:** Karissa Libson, Nina Mehta, Rachel Kirven, Abraham M. Korman, Benjamin H. Kaffenberger

**Affiliations:** 1 The Ohio State University College of Medicine, 2012 Kenny Road, Columbus, OH 43221 USA; 2 The University of North Carolina School of Medicine, Chapel Hill, NC USA; 3 Department of Dermatology, The Ohio State University Wexner Medical Center, Columbus, OH USA

**Keywords:** Epidermal necrolysis, Steven Johnson’s syndrome, Toxic epidermal necrolysis, Drug reaction, Outcome measurements, Evidence-based research, Clinical trials

## Abstract

Steven Johnson Syndrome (SJS) and Toxic Epidermal Necrolysis (TEN), grouped together under the terminology of epidermal necrolysis (EN), are a spectrum of life-threatening dermatologic conditions. A lack of standardization and validation for existing endpoints has been identified as a key barrier to the comparison of these therapies and development of evidenced-based treatment. Following PRISMA guidelines, we conducted a systematic review of prospective studies involving systemic or topical treatments for EN, including dressing and ocular treatments. Outcomes were separated into mortality assessment, cutaneous outcomes, non-cutaneous clinical outcomes, and mucosal outcomes. The COSMIN Risk of Bias tool was used to assess the quality of studies on reliability and measurement error of outcome measurement instruments. Outcomes across studies assessing treatment in the acute phase of EN were varied. Most data came from prospective case reports and cohort studies representing the lack of available randomized clinical trial data available in EN. Our search did not reveal any EN-specific validated measures or scoring tools used to assess disease progression and outcomes. Less than half of included studies were considered “adequate” for COSMIN risk of bias in reliability and measurement error of outcome measurement instruments. With little consensus about management and treatment of EN, consistency and validation of measured outcomes is of the upmost importance for future studies to compare outcomes across treatments and identify the most effective means of combating the disease with the highest mortality managed by dermatologists.

## Introduction

Steven Johnson Syndrome (SJS) and Toxic Epidermal Necrolysis (TEN) are grouped together under the terminology of epidermal necrolysis (EN), which is a life-threatening dermatologic condition characterized by a dusky morbilliform eruption with mucosal involvement, that rapidly progresses to vesicles, blisters, and skin sloughing.

There is a well-established need for evidence-based studies to define best supportive care practices and short-term treatment in EN [1]. Most existing studies examining these treatment methods are retrospective, but several randomized clinical trials have been completed. A lack of standardization and validation for existing endpoints has been identified as a key barrier when comparing therapies, hindering the development of evidenced-based treatment [1]. The purpose of this review is to systematically evaluate instruments used for outcome measures in prospective studies in the acute phase of EN.

## Methods

This systematic review followed PRISMA guidelines and was registered in PROSPERO (CRD42023405479). PubMed and EMBASE were utilized to identify prospective studies evaluating systemic and/or topical treatments for EN, including dressings and ocular treatments. We included studies which used one or more acute, EN-specific therapies, reported clear outcome measurements, were prospective in nature, and were available in English. Studies were excluded if they were not primary literature, presented outcome measurements for EN that could not be separated from other conditions, or assessed outcome measures related to EN secondary complications (e.g. xerophthalmia).

Data collected from these studies included study type, outcome measures, and primary or secondary endpoints if defined. The Cochrane Risk of Bias 2 (RoB 2) tool was used to assess the risk of bias in randomized control trials (RCTs) [2]. Each study was reviewed and scored according to the recommended protocol given by the authors of the tool.

## Results

**Fig. 1:**
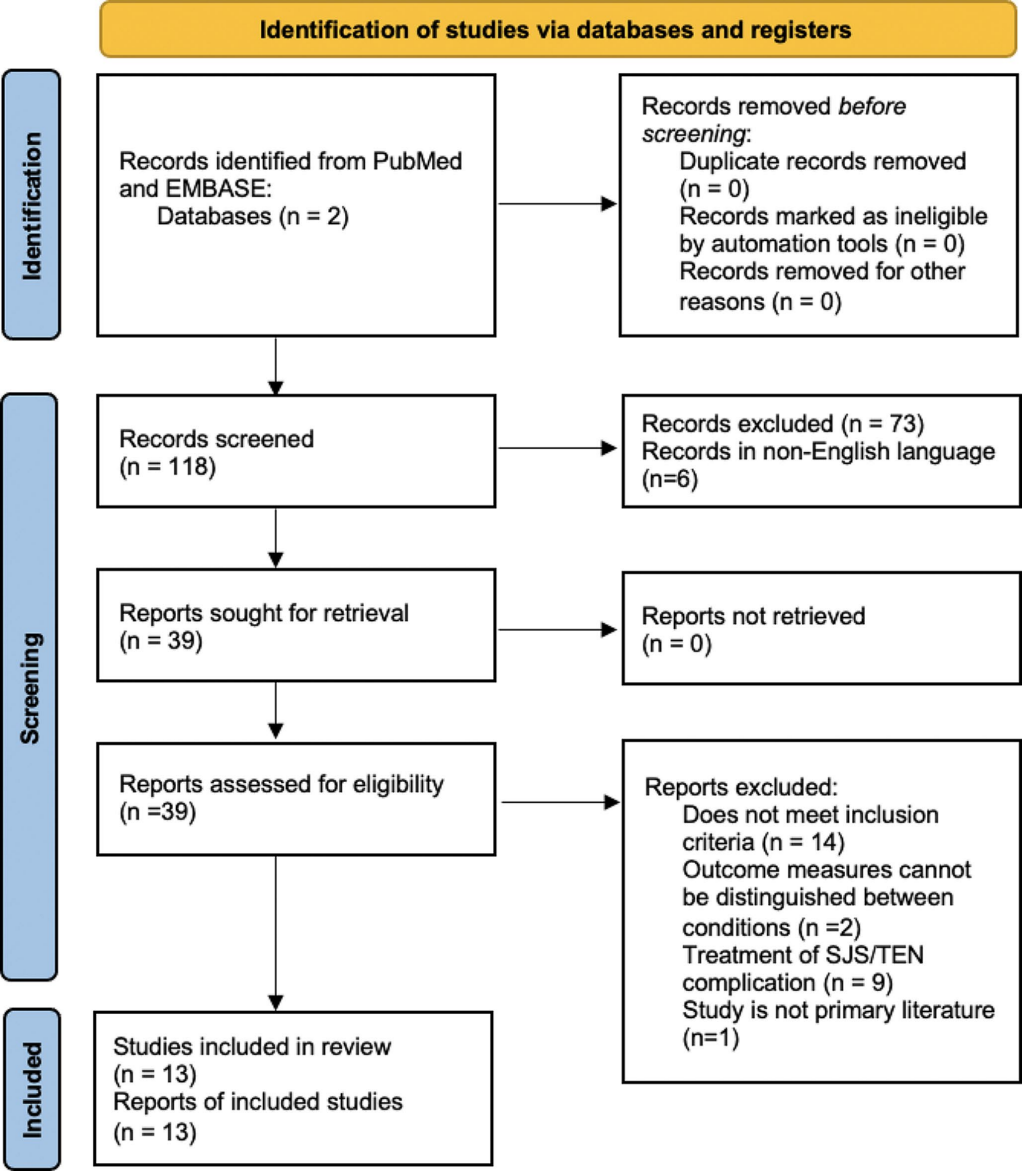
PRISMA flow diagram for identifying studies assessing treatment of acute EN

A total of 13 studies from 1997 to 2018 were included in the analysis (Fig. 1). Three RCTs, 5 case series, and 5 retrospective cohort studies were identified. Outcomes measures were divided into four categories: mortality assessment, cutaneous outcomes, mucosal outcomes, and non-cutaneous outcomes (Table 1).

**Table 1:** Outcome measurements in treatment of acute EN

Mortality Assessment			
Instrument	Range	Description	Study
Alternate treatment and retrospective comparison	N/A	Mortality from treatment group compared with other experimental group and retrospective database of patients who received supportive care.	Wang [4]
Placebo control comparison	N/A	Mortality from treatment group compared vs. placebo control.	Wolkenstein [5]
			Paquet [6]
			Paquet [7]
Standardized mortality ratio	N/A	Ratio of observed deaths to expected deaths calculated using SCORTEN.	Wang [4]
			Singh [8]
			Bachot [9]
			Valeyrie-Allanore [10]
**Cutaneous Outcomes**			
**Outcome**	**Range**	**Description**	**Study**
Acute eruption duration	0 to unlimited (worse)	Acute eruption was defined as the time from eruption onset to resolution characterized by red-blue color developing at the margins of the cutaneous lesions.	Kakourou [11]
Erythema extent	0-100%(worse)	The extent of erythema was measured on the assumption that the patient’s palm (including fingers) was equivalent to 1% of the body surface area. The pigmented areas were excluded.	Aihara [3]
Skin detachment extent	0-100%(worse)	The extent of epidermal detachment was measured on the assumption that the patient’s palm (including fingers) was equivalent to 1% of the body surface area. The epithelialized areas were excluded.	Aihara [3]
		Erosions, blisters, and areas with positive Nikolsky sign were measured at admission, on day 3, and on day 11 and expressed as a percentage of the TBSA using classic burn tables. Change was considered meaningful if it was greater than 2% for patients with an initial involvement of less than 10%, 5% or more for patients with an initial involvement between 10% and less than 30%, and 10% or more for patients with initial involvement greater than 30%.	Bachot [9]
Skin detachment progression	0-100%(worse)	Comparison of skin detachment percent between day 0 and 7	Wolkenstein [5]
		Progression of skin detachment between admission and day 3, evaluated daily as the percentage of BSA with blisters, erosions, and positive Nikolsky sign using classic burn tables. as meaningful if it was greater than 2% for patients with an initial involvement of < 10% at admission, 5% or more for patients with an initial involved BSA between 10% and 29%, and 10% or more for patients with an initial involved BSA > 30%.	Valeyrie-Allanore [10]
	0 to unlimited (worse)	Time in days between onset of IVIG treatment and interruption of further epidermal detachment.	Viard [12]
Skin healing duration	0 to unlimited (worse)	Time in days to skin healing.	Wang [4]
			Bachot [9]
Skin reepithelialization commencement	0 to unlimited (worse)	Time in days at which the reepithelialization of the denuded area commenced.	Wang [4]
Skin Reepithelialization extent	0-100%(better)	Percent of skin reepithelialized at day 5.	Paquet [6]
**Mucosal Outcomes**			
**Outcome**	**Range**	**Description**	**Study**
Adverse events or complications	Presence or absence	Description of complication or adverse event.	Sharma [13]
Oral mucosa healing duration	0 to unlimited (worse)	Time in days to healing of oral mucosa.	Wang [4]
Tear break up time (TBUT) changes	0 to unlimited (better)	TBUT measured before intervention and at follow up.	Sharma [13]
Visual Acuity changes	Worse, same, improved	Best corrected visual acuity (BCVA) measured before intervention and at follow up. Determined to be worse, same, or improved.	Sharma [13]
**Non-cutaneous Clinical Outcomes**			
**Outcome**	**Range**	**Description**	**Study**
Adverse events and complications	Presence or absence	presence or absence, description of event or complication.	Kakourou [11]
			Wang [4]
			Paquet [6]
Fever duration	0 to unlimited (worse)	Time in days of temperature over 38 degrees Celsius.	Kakourou [11]
General state of patient following treatment	Recovery, resolution, or death	none provided.	Tripathi [14]
			Cheriyan [15]
Hospitalization time	0 to unlimited (worse)	Time in days of patient hospitalization.	Paquet [6]
			Valeyrie-Allanore [10]
			Singh [8]
Severity of illness Score changes	0–39 (worse)	Rating scale that scores ophthalmic lesions, lip/oral lesions, cutaneous lesions, and general condition (oral intake, malaise, fever), with a total score ranging 0–39. Based on the results of a retrospective survey using the severity-of-illness score, patients with a reduction of 6 or more from day 1 of the therapy were considered responders. Measured on day 1 and day 7 of treatment.	Aihara [3]
Simplified Acute Physiology Score (SAPS) changes	0-163 (worse)	SAPS is a prognosis score calculated from seven clinical variables (age, heart rate, systolic blood pressure, body temperature, respiratory rate, urinary output per 24 h, Glasgow coma score) and seven biological variables (blood urea, packed-cell volume, white blood cell count, and plasma concentrations of glucose potassium, sodium, and bicarbonate). The variations of SAPS were expressed as the difference in SAPS at days 5 and 7 versus day 0.	Wolkenstein [5]

### Mortality assessment

Seven studies assessed patient mortality. The most commonly used metric to assess mortality was the standardized mortality ratio (*n* = 4), which is defined as the ratio of observed deaths compared to the expected deaths using the severity of illness score for TEN (SCORTEN) mortality predictor and is a frequently utilized endpoint in studies of epidermal necrolysis [1]. Other methods used in mortality assessment included comparison of treatment group mortality to an alternate treatment group (*n* = 1), placebo group (*n* = 3), or historical control group from a retrospective database (*n* = 1, Table 1).

### Cutaneous outcomes

Assessment of cutaneous outcomes primarily focused on skin detachment and reepithelialization. Studies that quantified cutaneous involvement (*n* = 8) used classic burn tables or palm measurements to provide an estimated body surface area. All cutaneous outcomes were clinician reported outcome measurements (ClinROM) and there were no validated methods or instruments utilized.

### Mucosal outcomes

Outcomes of oral and/or ocular mucosal disease were assessed in 2 of 13 studies (Table 2). Measured ocular outcomes included adverse events and complications, changes in tear break up time (TBUT), and visual acuity. All mucosal outcomes were ClinROMs and were unvalidated.

**Table 2:** Risk of bias in randomized control trials using the cochrane risk of bias 2 (RoB 2 tool)

Cochrane RoB 2 Tool Domain	Wang 2018 [4]	Sharma 2016 [13]	Wolkenstein 1998 [5]
1. Randomization Process	Low	Low	Low
2. Deviation from Intended Intervention	Some concerns	Some concerns	Some concerns
3. Missing Outcome Data	Low	Low	High
4. Measurement of the Outcome	Some concerns	Some concerns	Some concerns
5. Selection of the Reported Result	Some concerns	Some concerns	Some concerns
Overall Risk of Bias	Some concerns	Some concerns	Some concerns

### Non-cutaneous clinical outcomes

Three studies collectively assessed non-cutaneous adverse events and complications, including hospitalization duration. Adverse events, complications, and general state of health, following treatment were non-standardized descriptive reports by authors.

Scoring tools used included the Severity of Illness Score (*n* = 1) and Simplified Acute Physiology Score (SAPS, *n* = 1). SAPS is a validated critical care scoring tool used to assess prognosis in critically ill patients and severity of disease in clinical trials. Scores were calculated and compared at different time points to quantify treatment effectiveness. In contrast, the Severity of Illness Score used by Aihara et al. was designed from a retrospective survey assessing ophthalmic lesions, lip/oral lesions, cutaneous lesions, and general condition [3]. Other objective outcomes included hospitalization time and fever duration. All overall clinical outcome measures were ClinROMs.

### Risk of bias in randomized control trials

The Cochrane RoB 2 tool was created to provide an assessment of the risk of bias in RCTs, where bias is defined as systematic deviation from a theoretically ideal RCT. All three RCTs included in this study had some concerns for risk of bias overall (Table 2). All studies had low risk of bias in the randomization process and two had low risk of bias due to missing outcome data. In contrast, one study had high rates of patient death resulting in a high risk of bias due to missing outcome data. There were concerns noted in every other domain for all studies.

## Discussion

Measured outcomes used to assess treatment effectiveness in the acute phase of EN varied across studies. Most data came from prospective case reports and cohort studies representing the lack of available randomized clinical trial data available in EN.

The search did not reveal any EN-specific validated measures or scoring tools used to assess disease progression and outcomes. Adverse events and complications were assessed in multiple studies; however, they were not a priori defined, and it was unclear if these events were the result of the EN disease process, therapeutics, or another cause.

The standardized mortality ratio was widely used across studies. As an outcome measurement, the standardized mortality ratio likely requires additional calibration as SCORTEN is thought to underestimate mortality for low scores and overestimate mortality for high scores [1]. Regardless, the standardized mortality ratio provides a consistent (albeit unvalidated) method of mortality assessment across studies.

Measures of EN-specific cutaneous outcomes across studies varied. Many studies assessed skin detachment or reepithelialization, however their specific endpoints were nuanced. Some studies took a “time-to-event” approach, which measures the time to desired outcome, such as completion or commencement of skin reepithelialization. This measurement does not include data from patients who died, and therefore must be considered in light of mortality measurements. Further, measurement of epithelial damage and healing has not been standardized as an outcome measure across clinicians.

In addition to the time-to-event approach, several studies compared cutaneous outcomes (skin detachment, reepithelialization) at predetermined timepoints. While EN is known to take weeks to resolve, there was no apparent strategic rationale for selecting these timepoints in relation to the SJS/TEN disease process. Further, there is no validated metric to quantitate morphology and distribution of cutaneous lesions at baseline for adequate comparison.

Interestingly, resolution of oral mucosal and ocular involvement were monitored in one study each [4, 5]. All outcomes measured were ClinROMs and there were no patient reported outcome measures (PROMs). There are currently no studies which assess the validity of ClinROMs or PROMs as outcome measurements for EN.

Across all RCTs included in this review, there was some concern for risk of bias. It is worth considering that the high mortality rate of patients in Wolkenstein et al. contributed to a high risk of bias from missing outcome data [5]. This highlights a unique challenge in minimizing bias for RCTs looking at treatments for EN, especially at tertiary centers which accept patients with increased disease severity.

Limitations of this study include the exclusion of non-prospective literature and studies in languages other than English.

With little consensus about management and treatment of EN, development of validated outcome measures, with representation of the oral and genital mucosa, is essential for future studies including RTCs. One way of accomplishing this is the establishment of a Core Outcome Set for EN, which identifies consensus-based outcomes to be measured and reported across all clinical trials.

## Data Availability

The data that support the findings of this study are available from the corresponding author upon reasonable request. This manuscript has not been previously published or presented.
